# Evaluation of Barriers to Audit-and-Feedback Programs That Used Direct Observation of Hand Hygiene Compliance: A Qualitative Study

**DOI:** 10.1001/jamanetworkopen.2018.3344

**Published:** 2018-10-12

**Authors:** Daniel J. Livorsi, Cassie Cunningham Goedken, Michael Sauder, Mark W. Vander Weg, Eli N. Perencevich, Heather Schacht Reisinger

**Affiliations:** 1 Center for Comprehensive Access Delivery & Research, Iowa City Veterans Affairs Health Care System, Iowa City, Iowa; 2 Department of Internal Medicine, Carver College of Medicine, University of Iowa, Iowa City; 3 Department of Sociology, University of Iowa, Iowa City; 4 Department of Psychological and Brain Sciences, University of Iowa, Iowa City

## Abstract

**Importance:**

Audit and feedback based on direct observation is a common strategy to improve hand hygiene compliance, but the optimal design and delivery of this intervention are poorly defined.

**Objective:**

To describe barriers encountered by audit-and-feedback programs for hand hygiene across acute care hospitals within the Veterans Health Administration.

**Design, Setting, and Participants:**

A qualitative study was conducted at a geographically diverse convenience sample of 10 acute care hospitals within the Veterans Health Administration. Participants included 108 infection prevention team personnel and frontline staff. All data were collected between June 30, 2014, and March 18, 2015. Data were analyzed between September 6, 2017, and January 5, 2018.

**Main Outcomes and Measures:**

Barriers to audit and feedback for hand hygiene compliance were evaluated. Semistructured interviews of key personnel were performed through site visits at 6 locations and telephone interviews with 4 sites. Focus groups were conducted with frontline staff. Interviews and focus groups were audio recorded and transcribed. All transcripts were analyzed using thematic content analysis.

**Results:**

Overall, 108 individuals participated in the study. Semistructured interviews were conducted with 38 individuals, who were predominantly infection prevention team members. Focus group interviews were conducted with 70 frontline hospital staff members. Surveillance activities at all 10 sites made use of a variety of staff members with the intention of covertly collecting direct observations on hand hygiene compliance. Monitoring programs were challenging to maintain because of constraints on time and personnel. Both auditors and frontline staff expressed skepticism about the accuracy of compliance data based on direct observations. Auditors expressed concern about the Hawthorne effect, while frontline staff were worried that their compliance was not visible to auditors. In most hospitals, approaches to monitoring hand hygiene compliance produced friction between frontline staff and infection prevention teams. The feedback process for audit results did not consistently reach frontline staff and, in many hospitals, did not seem to facilitate improvement efforts.

**Conclusions and Relevance:**

Auditing hand hygiene compliance with direct observation was problematic across these acute care hospitals. Auditing was perceived to collect inaccurate data and created tension with frontline staff, and the feedback process did not appear to encourage positive change. Strategies are needed to collect more reliable hand hygiene data and facilitate multidisciplinary collaboration toward improved compliance.

## Key Points

**Question:**

How have audit-and-feedback programs based on direct observations of hand hygiene compliance been implemented in real-world settings?

**Findings:**

In this qualitative study of 108 hospital staff members in 10 acute care hospitals, the use of audit and feedback to improve hand hygiene compliance was problematic. Auditing by direct observation was perceived to collect inaccurate data and created tension with frontline staff, and the feedback process did not appear to encourage positive change.

**Meaning:**

Strategies are needed to collect more reliable hand hygiene data and facilitate multidisciplinary collaboration toward improved hand hygiene compliance.

## Teaser

This qualitative study examines the use and barriers to use of audit-and-feedback programs that use direct observation to assess hand hygiene compliance in acute care hospitals.

## Introduction

Hand hygiene is widely recognized as the most effective practice for preventing health care–associated infections, but rates of hand hygiene compliance among health care workers (HCWs) are only 34% to 57%.^1^

One strategy for improving hand hygiene compliance is audit and feedback. Audit and feedback is a strategy based on the premise that HCWs will be motivated to change their behavior when shown how their current performance differs from a stated goal. For many professional practices, including hand hygiene compliance, the use of audit and feedback can lead to small but measurable improvements in performance.^2,3,4,5,6^ Even small improvements in hand hygiene compliance can help to prevent health care–associated infections.^7,8^

Several guidelines and regulatory bodies recognize the importance of audit and feedback to hand hygiene improvement efforts. For example, guidelines from the World Health Organization,^9^ the Centers for Disease Control and Prevention,^10^ and the National Health Service in England^11^ all recommend using audit and feedback to improve hand hygiene compliance but stop short of providing specific guidance on how audit and feedback should be conducted. In addition, The Joint Commission’s Hospital Accreditation Program mandates that each hospital “monitors compliance [with hand hygiene] and provides feedback.”^12^^(p3)^

Given these recommendations and regulations, it is not surprising that audit and feedback is frequently used to improve hand hygiene compliance. In a 2012 survey of 141 medical centers within the Veterans Health Administration (VHA), nearly all facilities reported conducting direct observations of hand hygiene compliance and providing some form of feedback to leadership, units, and/or specific individuals.^13^

Audit and feedback can be implemented in a number of different ways. While certain strategies have been shown to improve the effectiveness of audit and feedback, the optimal design and delivery of these strategies are poorly defined.^2,14^ In this ethnographic study, we explored real-life barriers across VHA acute care hospitals to using audit and feedback based on direct observation of hand hygiene compliance.

## Methods

Our team conducted a qualitative study across 10 acute care hospitals within the VHA. This study was meant to assess baseline activities at all 10 sites prior to conducting a larger trial to improve hand hygiene compliance. At each site, a medical anthropologist (H.S.R.) and a social scientist (C.C.G.) performed semistructured interviews with hospital staff directly involved with hand hygiene surveillance and improvement efforts, including hospital epidemiologists, infection preventionists (IPs), multidrug-resistant organism prevention program coordinators (MDRO-PCs), and other quality and patient safety staff. Site visits were conducted at a geographically diverse convenience sample of hospitals, and interviews were performed in person at these 6 sites. At the remaining 4 sites, semistructured interviews were conducted via telephone. The semistructured interviews addressed past and current strategies to promote hand hygiene compliance, hand hygiene surveillance practices, and how these activities were structured at the facility (eAppendices 1-3 in the Supplement). During the site visits, the study team conducted focus groups with frontline staff (ie, nurses, physicians, unit clerks, and environmental service workers). The focus groups addressed frontline staff members’ knowledge and perceptions of strategies to promote and monitor hand hygiene compliance on their unit and at their facility. The study team also reviewed tools that each site used for hand hygiene surveillance and feedback. All data were collected between June 30, 2014, and March 18, 2015. Data were analyzed between September 6, 2017, and January 5, 2018.

The study adhered to the Consolidated Criteria for Reporting Qualitative Research (COREQ) reporting guideline (eAppendix 4 in the Supplement).^15^ The study was approved by the VA Central Institutional Review Board and Research and Development Committee at the Iowa City VA Health Care System. Verbal consent was obtained from all participants before interviews and focus groups were conducted. All participants gave permission for their quotations to be published anonymously; there was no financial compensation.

Interviews and focus groups were recorded and transcribed by trained staff. Transcriptions were reviewed against original recordings by the interviewers. All transcripts were imported into MAXQDA, version 12 qualitative data management software.

A thematic content analysis was performed on all transcripts.^16,17^ First, the analysis team reviewed 3 transcripts and developed a codebook, which drew on a priori research questions and emergent content. To increase validity and consistency in coding, 49% of the transcripts were coded by consensus at biweekly meetings with the analysis team (C.C.G., M.W.V.W., and H.S.R.) throughout the coding process. The remaining 51% of the transcripts were coded during this same period by 2 coders who initially coded independently and then met to reach consensus. In the next phase of the analysis, a new team (D.J.L., C.C.G., M.S., and H.S.R.) subcoded the 2 central themes of the original analysis: hand hygiene monitoring and hand hygiene intervention strategies. This article is based on the subcoding of these 2 themes.

## Results

Overall, 108 hospital staff members participated in the study, including semistructured interviews with 38 individuals and focus group interviews with 70 (Table 1). Based on audits performed by study personnel at 9 of the 10 sites, hand hygiene compliance rates were 49.8% (n = 9791) at room entry and 63.9% (n = 10 135) at room exit.

**Table 1.** Individual and Group Semistructured Interview Participants at 10 Veterans Health Administration Hospitals

Role	Participants, No.
**Semistructured Interview**	
Hospital epidemiologist	10
Infection preventionist	15
MDRO-PC	7
Other (eg, quality, patient safety)	6
Total	38
**Focus Group Interview**	
Nursing staff	53
Physician	3
Environmental services	3
Administrative	3
Other	7
NR	1
Total	70

Abbreviations: MDRO-PC, multidrug-resistant organism prevention program coordinator; NR, no response.

### Description of Audit-and-Feedback Activities at 10 Sites

All 10 sites were collecting direct observations to monitor hand hygiene compliance. The standard goal was 10 to 30 direct observations per unit per month. Audit results were typically aggregated at the unit level and at times broken down by disciplines (eg, physician, nurse).

Direct observations were collected by a team of individuals who could represent any of the following disciplines: IPs, MDRO-PCs, quality and patient safety staff, students, other nonclinical staff, and clinicians who did or did not work on the unit. Programs attempted to keep observers’ auditing role covert so staff did not know their hand hygiene behavior was being observed.

These internal monitoring processes consistently reported hand hygiene compliance rates that were substantially higher than the low compliance rates noted by study personnel (reported above). Additional information on hand hygiene surveillance activities can be found in the eTable in the Supplement.

The feedback of audit results was done in a variety of ways across the 10 sites. All monitoring programs reported their audit results to hospital leadership. To reach frontline staff, audit results were posted in break rooms, reported at unit meetings/huddles, and/or posted to an internal website. Only 1 site provided real-time feedback to noncompliers. Interviews revealed 4 primary themes about efforts to audit and provide feedback on hand hygiene compliance among HCWs (Table 2).

**Table 2.** Sample Quotations From Semistructured Interviews With Hand Hygiene Auditors and Focus Groups With Frontline Staff Across 10 Veterans Health Administration Hospitals

Category	Illustrative Quotation
Theme 1: Hand hygiene monitoring programs were challenging to maintain because of lack of time and personnel	“We’ve been so busy with these other things for the past month. I haven’t done observations in probably a month, which is terrible to say but it’s the truth. So we have horrible data.” (site 3, IP)
	“But, as we were discussing the barriers, you know, historically, infection control had always tried to run that and get people to send the monitors to us and, I mean, we were super creative and tried to get people involved and it just was like beating a dead horse.” (site 6, infection prevention director)
	“Sometimes you schedule an hour and you just say you’re going to go out, but then you get the phone call from the lab that you have a positive *Legionella*. You have some other crisis and then…the priority shifts.” (site 3, IP)
	“Because I think we’re asking a lot from the nursing personnel…Some of them, after a while, don’t even want to do it, because it’s extra.” (site 10, IP)
	“But since that [the ability to give incentives] has been taken away from us, our numbers have decreased and staff are not as willing to become observers for us. So we get secret shoppers…from volunteer services when available, but that’s sporadic. Most of our observations are from us going out.” (site 3, MDRO-PC)
	Success in recruiting other departments to assist with audits: “I eventually knew it was way too big for me to get everywhere in the hospital so I actually initiated and got departments involved in documenting the hand hygiene and that’s been a really big help.” (site 1, IP)
Theme 2: There was skepticism about the accuracy of hand hygiene monitoring data among both the auditors and those being audited	“Our observation programs are not reliable. And we believe that the compliance is far less than what the statistics may show.” (site 3, hospital epidemiologist)
	“We get a lot of feedback from other areas….especially EMS, the custodial staff, that they are constantly seeing people not washing….They do give us that feedback, but we can’t document it into the data unless we actually see it ourselves.” (site 8, MDRO-PC)
	“When you’re doing direct [observations] you pretty soon get tagged, and they know what you’re doing. That is a negative effect, because I think then people are running to wash their hands just because they see the person that’s documenting it.” (site 1, IP)
	“And it’s a huge dilemma. I mean, you’re out there and the minute they see you then they really start sanitizing in and out. Are those still valid observations even? We don’t think they are, because we saw at least as we were coming around the corner they weren’t complying and then they take a look at us and go, ‘Oh, got to wash my hands.’ Or they’ll see us walk in and 1 nurse will get up from the nurses’ station and go in to the room, you know, the ICU room where everybody is rounding, and say, ‘Oh, they’re out there observing. Wash your hands.’” (site 3, IP)
	“We still have the Hawthorne effect in play. Whenever the staff does see us, they automatically head towards the nearest either gel dispenser or sink.” (site 3, MDRO-PC)
	“The students [as covert observers] really for the first month are never known…but they [the frontline staff] quickly figure out who our students are. It’s not too hard.” (site 8, IP)
	“I think that there is no such thing as unobtrusive observation…I really think that if you’re really serious about it, you do what they did at Grand Island; they put in the cameras.” (site 10, hospital epidemiologist)
	“We can’t see behind the curtains and behind the doors. So that makes it a little difficult.” (site 3, MDRO-PC)
	“So you have to wash your hands in the room, and then you come out and there’s someone there. They catch you and say you didn’t wash your hands, because they don’t see you wash your hands.” (site 8, clinical nurse)
	“We realize that some of these percentages are based on very low numbers of observations. Like less than a dozen.” (site 1, hospital epidemiologist)
	“We realize we don’t get enough [observations]. I mean really that’s not a large enough sample to get a true picture of it. It’s the best we can do around here.” (site 6, IP)
	“There’s not enough observations, in my opinion…The ICU they did a project, and they had so many observations. That data is then really meaningful to us, but it’s hard to sustain because now who is going to do the audits? They have the rest of the work to do too.” (site 6, MDRO-PC)
Theme 3: Common approaches to monitoring hand hygiene compliance created tension between frontline staff and auditors	“We just call ‘em spies (laughter).” (site 9, clinical nurse)
	“Yeah, big brother’s watching (chuckling).” (site 2, clinician)
	“They really promote it when they have someone standing there with a clipboard giving you the evil eye. [laughter] I mean that’s a promotion. It might be in the negative form, but it’s still a promotion.” (site 6, clinical nurse)
	“We tried to deploy the iPods out [on the nursing units] and say [to the staff], ‘Hey you take the iPod for a shift and get as many observations as you want,’ and…they didn’t want to do observations for their unit. They didn’t want to quote unquote ‘narc’ on their coworker type of a thing. They really felt that it wasn’t their job.” (site 8, IP)
	“I encourage it [real-time feedback], but…there’s some strong people that [think] you have no right to say that. It’s the lack of team effort to work together to promote commonalities and better practice…There would be hostility if you would bring somebody to attention.” (site 9, clinical nurse leader)
	Positive examples of collaboration: “We used to do that [hand hygiene audits], but then we became the police, so we wanted it to go to a different group, so that we could focus on the interventions.” (site 6, infection prevention director) “It’s not like, ‘I’m glad we caught you.’ It’s more education, because you want compliance. You want people to buy into it, not to be like, ‘somebody’s watching me.’” (site 9, clinical nurse)
Theme 4: The feedback process for audit results did not consistently reach frontline staff and, in many hospitals, did not appear to motivate improvement efforts	“You send it up, but are they sending it back down? You’re the intermediary. You’re giving it up. How is it getting back down to the people?...Our responsibility really is to give it to leaders and for them to share it with their staff and develop their own action plans at the grassroots. However, once we tell the leaders…I should be able to go to the unit and say, ‘Hey, your hand hygiene rate was 64%. What are you—the nurse—gonna do about hand hygiene?’ and they should know that.” (site 9, IP)
	“We’re just gathering data, but we’re not using that to drive practice and make ourselves better.” (site 9, clinical nurse leader)
	“When I go and report these numbers to the quad, they really look at me with a deer-in-headlights look. Many times I don’t think that they understand what I’m talking about, so I really feel like I’m just showing them numbers. I really do. I never really come out of it with an action plan. It’s really kind of just sharing, say ‘Hey, here’s the numbers.’” (site 8, IP)
	“They probably put it up somewhere on the intranet, but most of us aren’t gonna work for it.” (site 6, nursing assistant)
	“We do post the hand hygiene compliance numbers for them to see. Probably becomes wallpaper, but you know.” (site 8, IP)
	“They were observers, and they were proactive, going to the person and letting them know [that hand hygiene wasn’t observed]….That started to increase the compliance rate….but then we got into another plateau where it doesn’t matter how many times you will come to the person to let them know they needed to wash their hands.” (site 9, patient safety nurse)

Abbreviations: EMS, emergency medical services; ICU, intensive care unit; IP, infection preventionist; MDRO-PC, multidrug-resistant organisms prevention program coordinator.

### Theme 1: Hand Hygiene Monitoring Programs Were Challenging to Maintain Because of Lack of Time and Personnel

There were many demands on individuals designated to collect direct observations on hand hygiene (ie, the auditors). For example, 1 IP said, “Sometimes you schedule an hour and you just say you’re going to go out [and perform direct observations for hand hygiene], but then you get the phone call from the lab that you have a positive *Legionella*. You have some other crisis and then…the priority shifts” (site 3).

Clinical staff assigned to collect direct observations experienced similar time constraints. According to 1 IP, “They [nurses] are in charge of doing patient care, and we’re asking them to do hand hygiene [audits]. And I know it creates a problem for some of them” (site 10).

Many IPs reported trying to recruit hospital staff members to assist with hand hygiene audits. While relying on staff to volunteer can help collect more direct observations, 1 hospital struggled with the high turnover rate among voluntary staff, which necessitated an ongoing need to train new staff volunteers (site 7). Other hospitals saw diminishing returns with volunteer recruitment efforts. At 1 site, the elimination of incentives had a detrimental effect on staff members’ willingness to volunteer as auditors: “Since that [the ability to give incentives] has been taken away from us, our numbers have decreased and staff are not as willing to become observers for us” (site 3, MDRO-PC). In contrast, 1 IP had been successful in recruiting volunteers: “I eventually knew it was way too big for me to get everywhere in the hospital, so I actually initiated and got departments involved in documenting hand hygiene, and that’s been a really big help” (site 1).

### Theme 2: Skepticism About the Accuracy of Hand Hygiene Monitoring

Skepticism about the accuracy of hand hygiene observation data was common across all sites. Staff involved in audits felt that directly observed compliance rates were artificially high, while clinical staff worried that their compliance to hand hygiene was often not visible to auditors. A major reason why the accuracy of audits was doubted was a general sense that frontline staff knew when they were being audited and therefore performed hand hygiene more frequently than usual. This phenomenon was frequently referred to as the Hawthorne effect. According to 1 IP, “When you’re doing direct [observation], you pretty soon get tagged, and they know what you’re doing. That is a negative effect, because I think then people are running to wash their hands just because they see the person that’s documenting it” (site 1). At times, individuals performing audits were even called out by clinical staff, thereby undermining the covertness of the audit: “They’ll see us walk in and 1 nurse will get up from the nurses’ station and go in to the room, you know, the [intensive care unit] room where everybody is rounding, and say, ‘Oh, they’re out there observing. Wash your hands’” (site 3, IP).

Even hospitals that have recruited nonhospital staff (eg, students) to perform audits have struggled with the Hawthorne effect. “The students really for the first month are never known…but they [the frontline staff] quickly figure out who our students are. It’s not too hard” (site 8, IP).

The difficulty in directly observing hand hygiene opportunities also contributes to skepticism about the accuracy of audit results. As 1 clinical nurse performing audits pointed out, “One hard thing is I can’t tell if maybe they’re using the pump [alcohol sanitizer] inside the room” (site 6). Clinical staff may be performing hand hygiene behind curtains or closed doors where they are beyond the view of auditors: “So you have to wash your hands in the room and then you come out and there’s someone there, they catch you and say you didn’t wash your hands, because they don’t see you wash your hands” (site 8, clinical nurse).

In general, auditors tried to avoid recording an HCW as noncompliant if these line-of-sight barriers were present. As a result, it was often challenging to gather even a small number of direct observations.

The small number of direct observations collected per month also fueled skepticism about the accuracy of audit results. At 1 hospital, the IP said, “We realize that some of these percentages are based on very low numbers of observations. Like less than a dozen” (site 1). An MDRO-PC also acknowledged that routine surveillance activities did not generate enough observations. In contrast, a multidisciplinary performance improvement project at this site’s intensive care unit collected several hundred observations per month, which was “meaningful.” This intensive care unit project, however, was short-lived: “It’s hard to sustain, because now who is going to do the audits? They have the rest of the work to do too” (site 6).

### Theme 3: Common Approaches to Monitoring Hand Hygiene Compliance Created Tension Between Frontline Staff and Auditors

At many sites, frontline staff frequently used negative terms to describe individuals involved in hand hygiene audits. Example terms included “spies” and “big brother.” A few frontline staff used a derogatory term to suggest that the auditors were being excessively strict.

Some auditors also sensed that they were working against, instead of with, the frontline staff they were auditing. Not only did auditors observe efforts to undermine the covertness of the audit, as discussed above, but some auditors sensed an unwillingness of frontline staff to collaborate: “It’s the lack of team effort to work together to promote commonalities and better practice....There would be hostility if you would bring somebody to attention [by using real-time feedback]” (site 9, clinical nurse leader). One IP speculated that this “us-vs-them” mentality contributed to her struggles to recruit frontline staff to assist with hand hygiene audits: “They didn’t want to quote unquote narc on their coworker type of a thing. They really felt that it wasn’t their job” (site 8).

A few IPs were able to navigate these tensions between the auditors and the frontline staff. For example, 1 group of IPs passed the responsibility for direct observations to another department: “We became the police [when we were doing audits], so we wanted it to go to a different group so that we could focus on the interventions” (site 6, IP director). At another site, a clinical nurse described how individuals who did not perform hand hygiene were approached, “It’s not like, ‘I’m glad we caught you.’ It’s more education, because you want compliance. You want people to buy into it, not to be like, ‘Somebody’s watching me’” (site 9).

### Theme 4: The Feedback Process for Audit Results Did Not Consistently Reach Frontline Staff or Motivate Improvement Efforts

Although all hand hygiene surveillance programs provided feedback on their audit results, it was unclear if this feedback was reaching the frontline staff. Many sites had created a dashboard on the intranet to report hand hygiene audit results, but several auditors had concerns about whether this process was effective. According to 1 hospital epidemiologist, the dashboard may be “too overwhelming for the user to make much sense out of [it]” (site 6). Another IP said, “The information is there, but like a lot of information, people might not know where to find it” (site 6). Several clinical nurses were unaware of their unit’s audit results. As 1 nursing assistant admitted, “They probably put it [the audit results] up somewhere on the intranet, but most of us aren’t gonna work for it” (site 6). At 1 site where audit results were posted in the nurses’ break room, an IP speculated that any given posting “probably becomes wallpaper” (site 8).

In general, individuals involved in hand hygiene monitoring felt that the feedback of audit results was not prompting the types of improvements they would hope to see: “We’re just gathering data but we’re not using that to drive practice and make ourselves better” (site 9, clinical nurse leader). An IP echoed this sentiment: “Our responsibility really is to give it [the audit results] to leaders and for them to share it with their staff and develop their own action plans at the grassroots. However, once we tell the leaders, …I should be able to go to the unit and say, ‘Hey, your hand hygiene rate was 64% percent. What are you—the nurse—gonna do about hand hygiene?’ and they should know that” (site 9).

## Discussion

Audit and feedback is widely recognized as a foundational strategy to improve hand hygiene compliance among HCWs.^18^ Our study has identified several barriers to the effective use of this approach.

Based on our findings, an essential step in improving the effectiveness of the audit-and-feedback process is establishing the credibility of direct observations for hand hygiene compliance. Performance data must be credible to drive behavior change,^14^ but in our study of real-life audit-and-feedback programs, the accuracy of direct observations was questioned. This skepticism was driven, in part, by concerns about the Hawthorne effect.^19^ That is, auditors were concerned that HCWs’ compliance to hand hygiene was higher than normal when the HCWs were being audited. Although all sites tried to covertly collect direct observations, our data suggest that auditors struggled to maintain their covert role. Prior studies have also shown that hand hygiene compliance is substantially higher in the presence of auditors, but at least based on 1 study, this result is not always a source of skepticism about audit results.^4,20,21,22^ To obtain more accurate compliance rates, evidence suggests that auditors should stay on units for no longer than 10 to 20 minutes at a time.^20,23,24^

In addition to the Hawthorne effect, skepticism about audits was driven by the perceived difficulty of observing HCWs in situations when hand hygiene is required. However, in a prospective study across 3 acute care hospitals, all entry-and-exit opportunities were visible to auditors, while only a third of the World Health Organization’s 5 Moments could be seen.^25^ Auditing solely based on entry-and-exit opportunities and educating health care professionals about the accuracy of this approach may improve the credibility of audit reports.

Our findings suggest that efforts to monitor hand hygiene compliance were perceived negatively by many frontline staff. It is unclear what has shaped these negative perceptions at the study sites and how they can be changed. It is possible that these negative sentiments reflect a failure to internalize hand hygiene norms and low levels of safety culture on the nursing units that we interviewed. In both a Dutch and US study, hospital units with low levels of safety culture were less open to new interventions to improve hand hygiene compliance.^26,27^ At a separate US hospital, units with the highest scores on 4 domains of the Safety Attitudes Questionnaire had significantly higher hand hygiene compliance rates than units with the lowest scores on those Safety Attitudes Questionnaire domains.^28^ Improving safety culture may be a prerequisite to increasing hand hygiene.

The existing literature offers several strategies for improving the feedback process, which was not perceived to be effective at participating sites.^14^ A Cochrane review of 49 randomized clinical trials found that 5 characteristics were associated with the improved effectiveness of feedback: (1) having a supervisor or senior colleague provide feedback, (2) providing feedback at least monthly, (3) providing feedback in both verbal and written forms, (4) using feedback to reduce instead of increase a particular behavior, and (5) setting clear goals with specific instructions for how to improve.^2^ Even though some of these features were part of audit-and-feedback activities at our study sites, most programs were not seeing results. This outcome may reflect a failure of implementation. In addition, there are opportunities to adopt some of the above-mentioned approaches, such as using supervisors to provide feedback or setting more specific, perhaps individualized, hand hygiene goals. A major knowledge gap is how to incorporate these and other features of effective audit and feedback into routine hand hygiene improvement programs.

More rapid, personalized feedback accompanied by goal setting has been used in at least 2 large intervention trials on hand hygiene.^4,29^ The first of these trials was a multicenter study across 16 hospitals, which achieved a sustained 7% to 9% improvement in hand hygiene compliance.^4^ The second trial was a single-center, cluster randomized trial in which hand hygiene compliance improved across both control and intervention groups, possibly owing to cross contamination.^29^ In addition, improved hand hygiene compliance was achieved at teaching hospitals in the United States and Switzerland through a multimodal intervention that included direct feedback when a hand hygiene opportunity was missed.^30,31^ All of these interventions required a substantial deployment of human resources, so their wider adoption may be challenging.

Technology may provide a means for delivering more rapid targeted feedback. For example, remote video surveillance with real-time group feedback improved hand hygiene compliance in an intensive care unit from 10% at baseline to more than 88% during a sustained intervention period.^6^ Several hospitals have used automated surveillance systems to provide immediate and individualized feedback that, in turn, has helped to improve hand hygiene compliance.^32,33,34,35^ While these automated systems may be an appropriate replacement or complement to direct observations, electronic systems present their own set of challenges, including HCWs’ resistance to being tracked and concerns about the accuracy of data capture.^36^ Furthermore, little research has been conducted on how to incorporate automated systems into an effective audit-and-feedback program, and automated systems cannot provide specific feedback on how HCWs are noncompliant. Until the cost of automated systems decreases, broader use of this technology will likely be limited.

### Strengths and Limitations

Our study has both strengths and limitations. To our knowledge, this evaluation represents one of the first natural inquiries into how audit and feedback has been implemented within acute care hospitals to improve hand hygiene compliance. Although we chose sites that were geographically diverse, the barriers described were common across the 10 sites. Since all sites were VHA hospitals, our findings may be less relevant to non-VHA and non-US facilities. Interviewees self-reported their processes, and we did not validate the accuracy of their statements. We did, however, collect hand hygiene compliance reports and policies from each site. Furthermore, even though all interviews were confidential, participants may have been inclined to give socially desirable responses.

## Conclusions

Auditing hand hygiene compliance using direct observation was problematic across these acute care hospitals. Performing audits was time-consuming, the audit results were not trusted, and the feedback process typically did not facilitate positive change. Strategies are needed to collect more reliable data and facilitate multidisciplinary collaboration toward improved compliance. Finally, greater incorporation of evidence-based interventions into audit-and-feedback activities for hand hygiene may improve their overall effectiveness.

## Notes

Supplement. **eAppendix 1.** Semistructured Interview Guide **eAppendix 2.** Focus Group Guide **eAppendix 3.** Observation Guide on Units **eAppendix 4.** Consolidated Criteria for Reporting Qualitative Studies (COREQ) **eTable.** Description of Hand Hygiene Surveillance Activities at 10 VHA Acute-Care Hospitals
