# Supplementary material for: Evaluation of Barriers to Audit-and-Feedback Programs That Used Direct Observation of Hand Hygiene Compliance: A Qualitative Study
Source: JAMA Netw Open. 2018 Oct 12;1(6):e183344. doi: 10.1001/jamanetworkopen.2018.3344 (PMC6324430; doi:10.1001/jamanetworkopen.2018.3344)
Supplement: Supplement. — eAppendix 1. Semistructured Interview Guide eAppendix 2. Focus Group Guide eAppendix 3. Observation Guide on Units eAppendix 4. Consolidated Criteria for Reporting Qualitative Studies (COREQ) eTable. Description of Hand Hygiene Surveillance Activities at 10 VHA Acute-Care Hospitals [file jamanetwopen-1-e183344-s001.pdf]

Supplementary Online Content

Livorsi DJ, Cunningham Goedken C, Sauder M, Vander Weg MW, Perencevich EN, Schacht Reisinger HS. Evaluation of barriers to audit-and-feedback programs that used direct observation of hand hygiene compliance. *JAMA Netw Open*. 2018;1(6):e183344. doi:10.1001/jamanetworkopen.2018.3344

eAppendix 1. Semistructured Interview Guide

eAppendix 2. Focus Group Guide

eAppendix 3. Observation Guide on Units

eAppendix 4. Consolidated Criteria for Reporting Qualitative Studies (COREQ)

eTable. Description of Hand Hygiene Surveillance Activities at 10 VHA Acute-Care Hospitals

This supplementary material has been provided by the authors to give readers additional information about their work.

eAppendix 1. Semi-Structured Interview Guide

These interviews will be conducted with the Infection Control (IC) teams at each of the five facilities at which we conduct site visits. Approximately 3 interviews will be conducted at each site, one with the Hospital Epidemiologist, one with the Infection Control Professional (ICP), and one with the MRSA/MDRA Coordinator. A modified version of this interview will be conducted over the phone with the ICP at the other five sites after the completion of the site visits.

1) What is the make-up of your IC team?
2) How long have you been in your position? Others on the team?
3) Do you know how the MRSA/MDRO position was integrated into the team after the mandate?
4) What are your current strategies for promoting hand hygiene compliance?
   Probes: signs, other reminders (e.g., screen savers), educational programs, improving accessibility of hand sanitizer, audit/feedback, one-on-one coaching, use of champions, incentives or rewards, punishment, patient engagement.
5) What has worked particularly well? In what ways?
6) What have you tried that hasn't worked? What got in the way of success?
7) Overall, how would you describe hand hygiene compliance at your facility?
   Examples: everyone is onboard, it is a major priority, it is abysmal, getting staff to wash their hands is the worse part of my job.
8) Do you have a written hand hygiene policy for your facility? Do you mind sharing it with me? Which staff in the facility are aware of this policy—or pieces of it?
9) How do you measure hand hygiene compliance?
10) What is your hand hygiene compliance goal?
11) Talk with me more about how you collect your hand hygiene data.
    a. If observation:
       i. Do you train staff? How?
       ii. Do you validate their observations? How? How often?
       iii. What are the numerator and denominator?
    b. If product usage:
       i. How do you collect product volume?
       ii. What facility staff help in the process?
       iii. What are the numerator and denominator?
12) Do you feed compliance data back to staff?
    a. How? To whom? Individualized? By unit?
13) What is leadership's role in hand hygiene at your facility?
14) What is your role?
15) The Joint Commission hand hygiene goals states that facilities should have a plan in place when compliance rates are not met. What is your plan when a unit falls below a particular threshold?
16) What else should we know about hand hygiene here? What have we forgot to ask about? Do you have any questions for me?

© 2018 Livorsi DJ et al. *JAMA Network Open.*

eAppendix 2. Focus Group Guide

All healthcare workers (nurses, physicians, respiratory therapists, dieticians, etc.) available on a unit at the time of the focus group will be invited to join. An invitation will be sent out to floor staff prior to the focus group so they are aware of it occurring. We will conduct 2 focus groups on different wards/units at each of five VA facilities.

1) Overall, how would you describe hand hygiene compliance at your facility?
   Examples: everyone is onboard, it is a major priority, it is abysmal, getting staff to wash their hands is the worse part of my job.
2) What are the current strategies for promoting hand hygiene compliance at your facility? Have you noticed: signs, other reminders (e.g., screen savers), educational programs, improved accessibility of hand sanitizer, audit/feedback, one-on-one coaching, use of champions, incentives or rewards, punishment, patient engagement?
3) What is the best hand hygiene intervention you can think of? Why was it so good?
4) What have you seen tried that hasn't worked? What got in the way of success?
5) Do you have a written hand hygiene policy for your facility? Do you know what it talks about?
6) Is hand hygiene compliance measured at your facility?
7) Do you get feedback on how you are doing? Or your unit?
8) What is your hand hygiene compliance goal?
9) Who is responsible for hand hygiene at your facility?
10) What if you were in charge of improving hand hygiene rates? What would you do?
11) Is leadership aware of hand hygiene compliance rates at your facility? Are they involved in hand hygiene interventions? Should they be?
12) What else should we know about hand hygiene here? What have we forgot to ask about? Do you have any questions for me?

eAppendix 3. Observation Guide on Units

1) Note number of hand hygiene posters, location (near point of use, in halls, in patient rooms, on beds, etc.), and source (VA IDPIO, CDC, etc.).

2) Note if hand hygiene compliance rates are displayed on unit. Location of display. Is the location in view of patients and families?

3) Note where sanitizer dispensers are located. Number of dispensers.

4) Note any infection control material on ward/unit. For example, indication of contact precaution rooms.

5) General observation of hand hygiene practices. (No need to count since this is part of the larger study.)

6) Are there any signs of the interventions continuing on the wards? Study signs hanging up? Use of individual hand sanitizer? Hand cultures displayed?

7) [When rounding with someone on IC team] Note ICP's general practice. For example, does he or she stop a HCW and talk with him or her about a missed hand hygiene opportunity? Does she check dispensers to make sure they are adequately filled?

© 2018 Livorsi DJ et al. *JAMA Network Open.*

eAppendix4. Consolidated criteria for reporting qualitative studies (COREQ): 32-item checklist[1]

No. Item	Guide questions/description	Reported on Page #
**Domain 1: Research team and reflexivity**		
Personal Characteristics		
1. Interviewer/facilitator	HSR and CCG conducted all interviews.	Methods
2. Credentials	HSR has a PhD in Anthropology. CCG has a Master's in Public Health. MS has a PhD in Sociology. MV has a PhD in Psychology. DJL and ENP are physicians with a Master's in Clinical Investigation.	Title page
3. Occupation	Medical anthropologist (HSR), Sociologist (MS), Psychologist (MV), Qualitative analyst (CCG), Physician (DJL, ENP)	Methods
4. Gender	Female and male	N/A
5. Experience and training	HSR a PhD-trained medical anthropologist. CCG has a MPH and has participated in multiple qualitative research projects. MS is a PhD-trained sociologist, and MV is a PhD-trained psychologist. ENP and DJL are physicians specializing in infectious diseases.	Not Included
Relationship with participants		
6. Relationship established	Relationships were limited to interviews.	Not Included
7. Participant knowledge of the interviewer	Prior to interviews, HSR and CCG provided general descriptions of the project goals and the purpose of the interviews, as well as a brief personal introduction, including affiliation, occupation, and training.	Not Included
8. Interviewer characteristics	No interviewer characteristics were reported.	Not Included
**Domain 2: study design**		
Theoretical framework		
9. Methodological orientation and Theory	Our approach is informed by Strauss and Corbin's (1998) refinement of grounded theory, which is attentive to how participants make sense of phenomena while also recognizing the value of incorporating literature into the analytic	Not Included

© 2018 Livorsi DJ et al. *JAMA Network Open.*

	process.	
Participant selection		
10. Sampling	We contacted all 10 sites and interviewed individuals directly involved in hand hygiene at their facility, as well as interviewed a convenience sample of frontline staff.	Methods
11. Method of approach	Email	Not included
12. Sample size	108 total completed interviews	Results and Table 1
13. Non-participation	None declined to participate	Not included
Setting		
14. Setting of data collection	Workplace and by telephone during work hours	Methods
15. Presence of non-participants	No	Not included
16. Description of sample	Both phone and in-person interviews were conducted with individuals directly involved with hand hygiene (e.g., Hospital Epidemiologists). In-person interviews were conducted with frontline staff (e.g., clinicians).	Methods and Results
Data collection		
17. Interview guide	We used an interview guide, including questions and prompts. The guide was not pilot tested.	Methods
18. Repeat interviews	For this paper, there were no repeat interviews.	N/A
19. Audio/visual recording	Interviews were audio recorded.	Methods
20. Field notes	Field notes were not included in this analysis.	N/A
21. Duration	Interview range = 6:47 minutes – 85:56 minutes	Not included
22. Data saturation	We reached thematic saturation at the level of 'hand hygiene audit' and 'hand hygiene feedback'	N/A
23. Transcripts returned	No	N/A
**Domain 3: analysis and findings**		
Data analysis		
24. Number of data coders		Methods
25. Description of the coding tree	We include the codes we analyzed for this manuscript, as well as providing a general discussion of how we developed codes	N/A

© 2018 Livorsi DJ et al. *JAMA Network Open.*

	from reoccurring ideas. We do not include a coding tree.	
26. Derivation of themes	Themes based on interview guide domains were identified *a priori*, and other themes emerged after coding interviews.	Methods
27. Software	MAXQDA v.12	Methods
28. Participant checking	No	Not Included
Reporting		
29. Quotations presented	Participant quotations are identified by role and site number.	Results
30. Data and findings consistent	Yes	Results
31. Clarity of major themes	Major themes are identified by subheadings in the Results section, and their relevance addressed in the Discussion.	Results and Discussion
32. Clarity of minor themes	While divergent perspectives are represented throughout, our primary goal was to be broad or comprehensive in our presentation of contextual factors influencing uptake.	Results and Discussion

[1]Tong A, Sainsbury P, Craig J. Consolidated criteria for reporting qualitative research (COREQ): a 32-item checklist for interviews and focus groups. *International Journal for Quality in Health Care*. 2007. Volume 19, Number 6: pp. 349 – 357

eTable. Description of Hand Hygiene Surveillance Activities at 10 VHA Acute-Care Hospitals		
**Who manages the hand hygiene surveillance program?**	Infection Prevention Team	6
	Quality/Patient Safety	4
**Who conducts direct observations of hand hygiene?**	Infection Prevention Team	4
	Quality/Patient Safety staff	2
	Unit-based nurse champions	7
	Other	2
**How is audit data collected?**	Paper/pencil	9
	iScrub	1
**Which hand hygiene opportunities are audited?**	Entry/Exit only	6
	WHO's 5 Moments	2
	Unique combination	2
**Documentation of reasons for non-compliance with hand hygiene**	Yes	3
	No	7
**Who enters audit results into the surveillance database?**	Infection Prevention Team	5
	Quality/Patient Safety Staff	3
	Unit-based nurse champions	1
	Automated	1
**Who reports auditing data to hospital leadership?**	Infection Prevention team	10

© 2018 Livorsi DJ et al. *JAMA Network Open.*
